# Real-world disease-modifying therapy pathways from administrative claims data in patients with multiple sclerosis

**DOI:** 10.1186/s12883-022-02738-7

**Published:** 2022-06-07

**Authors:** Robert J. Fox, Rina Mehta, Timothy Pham, Julie Park, Kathleen Wilson, Machaon Bonafede

**Affiliations:** 1 Mellen Center for Multiple Sclerosis, Cleveland Clinic, Cleveland, OH USA; 2 Bristol Myers Squibb, Princeton, NJ USA; 3 IBM Watson Health, 75 Binney Street, Cambridge, MB USA

**Keywords:** Treatment pathway, Sankey diagram, Treatment patterns, Administrative claims database

## Abstract

**Background:**

Over a dozen disease-modifying therapies (DMTs) have been approved for treatment of multiple sclerosis (MS). Treatment guidelines focus on when to initiate, change, and discontinue treatment but provide little guidance on how to select or sequence DMTs. This study assessed sequencing patterns of DMTs in patients with newly diagnosed MS.

**Methods:**

Adults newly diagnosed with MS in the United States were identified from January 2007 to October 2017 using IBM MarketScan database. Patients had ≥12 months of continuous enrollment prior to diagnosis and ≥ 2 years of follow-up. Treatment pathways consisting of up to 3 DMT courses were reported, and each treatment course ended with discontinuation, switch, or end of follow-up.

**Results:**

In total, 14,627 MS patients were treated with DMTs and had ≥2 years of follow-up. More than 400 DMT treatment pathways were observed. Glatiramer acetate was the most common DMT; 40% of patients initiated this treatment. Among these, 51.3% had 2 DMT courses during follow-up and 26.5% had 3 DMT courses. Approximately 70% of patients switched or discontinued their initial DMT, and rates of switch and discontinuation differed by initial DMT. Injectable DMTs were used most commonly over the study period (87.5% as first course to 66.6% as third course). Oral DMTs were more common as second or third treatment courses (29.9% and 31.8%, respectively).

**Conclusions:**

A wide variety in treatment patterns were observed among patients newly diagnosed with MS. Further examination of DMT prescribing practices is needed to understand the reasons behind treatment discontinuation and treatment cycling.

**Supplementary Information:**

The online version contains supplementary material available at 10.1186/s12883-022-02738-7.

## Background

Multiple sclerosis (MS) is a chronic and potentially disabling disease of the central nervous system that causes demyelination and axonal transection, resulting in progressive disability [1]. MS has no known cure, but treatment with disease-modifying therapies (DMTs) can alter the course of the disease [2–4]. As the majority of patients are diagnosed in early adulthood (age 20 to 40 years) [1], treatment and disease management need to consider the immediate needs of the individual patient while also focusing on strategies that prevent relapses and slow progression in the long-term.

While more than a dozen DMTs have been approved by the US Food and Drug Administration (FDA) for the treatment of the relapsing forms of MS, these medications differ in their routes of administration (injectable, oral, or infused), mechanisms of action, efficacy, risk profiles, and monitoring requirements [3, 5]. US treatment guidelines focus on when to initiate treatment, when to switch DMTs, and when to stop a DMT, but they provide limited guidance on how to select or sequence DMTs [6]. As a result, clinicians have significant latitude when selecting treatment pathways to best meet the needs of individual patients.

The objective of this study was to characterize trends in the treatment of MS by identifying the most common pathways of DMT treatment used by US patients newly diagnosed with MS.

## Methods

### Study design and data source

This investigation was an administrative claims–based study of DMT treatment pathways among US adults newly diagnosed with MS in the IBM MarketScan Commercial Claims and Encounters Database and the Medicare Supplemental and Coordination of Benefits Database [7]. The study used data generated from January 1, 2006, to March 31, 2018. The commercial database captures the inpatient medical, outpatient medical, and outpatient prescription drug data for more than 155 million employees and their dependents covered by a variety of fee-for-service and managed care health plans. The Medicare database contains the same type of data for approximately 10.6 million Medicare-eligible retirees covered by employer-sponsored Medicare Supplemental plans.

All study data were identified using codes from the International Classification of Diseases, Ninth and Tenth Revisions, Clinical Modification (ICD-9-CM and ICD-10-CM), the Current Procedural Terminology Fourth Edition, the Healthcare Common Procedure Coding System, and the National Drug Codes. All database records were statistically de-identified and certified as fully compliant with US patient confidentiality requirements set forth in the Health Insurance Portability and Accountability Act of 1996. Because this study used only de-identified patient records and did not involve the collection, use, or transmittal of individually identifiable data, administrative permissions were not required.

Data used in this analysis are from claims databases which utilizes deidentified insurance claims which would not require institutional review board approval as it does not fall within the regulatory definition of research involving human subjects.

### Patient/record selection

Patients with ≥2 non-diagnostic claims (ie, excluding claims, such as radiology or laboratory claims, associated with procedures that may be related to an attempt to rule out a condition) at least 1 day and no more than 365 days apart with a diagnosis of MS between January 1, 2007, and October 1, 2017, and no claims with an MS diagnosis in the 12 months preceding the first eligible MS claim, were identified in the MarketScan Commercial and Medicare databases. Previous studies validated this selection methodology to identify MS patients from administrative databases [8]. The index date was the date of the first eligible MS claim. Eligible patients were required to have ≥12 months of continuous enrollment with healthcare and pharmacy benefits before the index date and 2 years after the index date. The 2-year follow-up enrollment requirement was used so there would be sufficient follow-up time to observe longitudinal changes in DMT for each patient. In addition, patients younger than 18 years on the index date or with evidence of pregnancy or primary malignancy anytime during the study period were excluded.

Patients were followed for a minimum of 2 years until the earliest of the following: inpatient death, end of continuous enrollment, or end of the study period (March 31, 2018). The current analysis focuses on the subset of patients who initiated an eligible DMT after their MS diagnosis and who had only a single DMT on the date of their first DMT claim. This study included injectable (glatiramer acetate, intramuscular interferon beta-1a [IFN β-1a IM], subcutaneous interferon beta-1a [IFN β-1a SC], interferon beta-1b [IFN β-1b], and peginterferon beta-1a [pegIFN β-1a]), oral (dimethyl fumarate, fingolimod, and teriflunomide), and infusion (natalizumab and ocrelizumab) DMTs. The MS treatments alemtuzumab and mitoxantrone were not included in this analysis owing to late approval date or low utilization based on a preliminary analysis of the dataset.

### Patient characteristics

Baseline patient demographics were measured on the index date and included age, sex, geographic region, index year, and duration of follow-up.

### Treatment patterns and pathways

All DMTs administered to patients with MS were described over the variable-length follow-up period of 2–10.5 years and reported by continuous treatment course for up to 3 courses. All patients had ≥1 treatment course, which started on the date of the first DMT medical or pharmacy claim after the index date. The treatment course continued until the earliest of the following: switch, discontinuation, or end of follow-up (ie, inpatient death, end of continuous enrollment, or end of the study period). Switching was defined as the initiation of a new DMT. Discontinuation was defined as a gap of ≥60 days after the end of supply of outpatient pharmacy prescriptions or by dosing schedule for medications administered in the office. This definition of discontinuation, based on a ≥ 60 day gap in therapy, has been used in previous studies [9–11].

For patients with subsequent treatment courses (ie, a second and/or third), the course start date was defined as the date of the first DMT after a treatment switch or the discontinuation of the previous treatment course. Treatment course end dates were determined using the same criteria as the first treatment course. Restarting a DMT after a gap in treatment of ≥60 days was considered a new treatment course. For each treatment course, the following outcomes were measured: DMT used, days to start of treatment course, duration of treatment course, and whether the treatment course ended due to switch, discontinuation, or end of follow-up. Treatment pathways included up to 3 DMT treatment courses. The duration of time on the treatment pathway included the time on the DMT treatment course as well as any gaps between courses; whereas, the time on drug included the time on DMT only.

### Statistical analyses

Continuous variables were reported as mean and standard deviation (SD). Categorical variables were reported as the number and proportion of patients. All data analyses were conducted using WPS version 4.1 (World Programming, UK). A Sankey diagram of all treatment pathways followed by ≥50 patients was prepared using R version 3.5.1 (R Foundation for Statistical Computing, Vienna, Austria).

## Results

### Patient characteristics

**Fig. 1:**
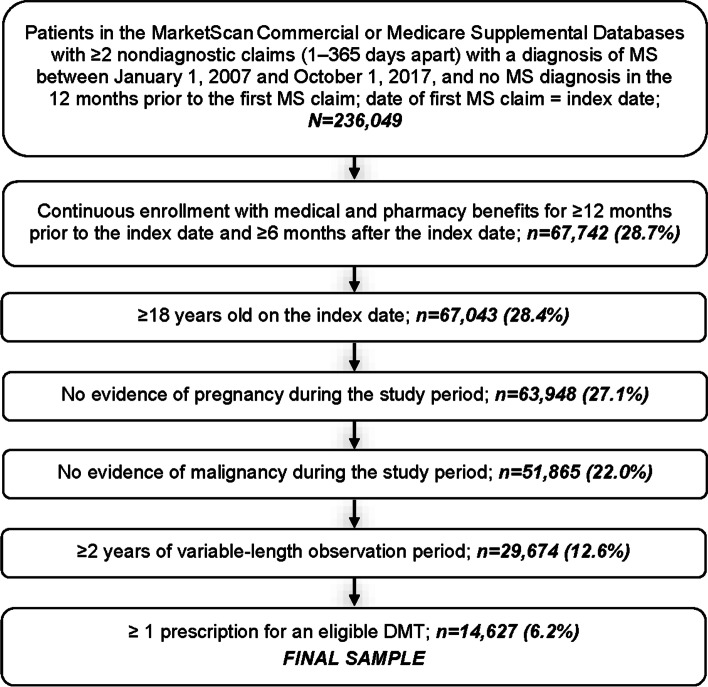
Patient attrition

Of 29,647 patients newly diagnosed with MS who had ≥2 years of follow-up data, 14,627 (49.3%) were treated with DMTs and 15,020 (50.7%) were not treated during the variable-length follow-up period (Fig. 1). The mean ± SD age of DMT-treated patients was 47.4 ± 11.0 years and 73.5% of patients were female (Table 1). For comparison, untreated patients were older at index (53.8 ± 14.3 years) and a similar percentage was female (72.5%; Supplementary Table 1).

**Table 1:** Patient characteristics

	DMT-treated patients ***N*** = 14,627	
**Age (mean, SD), y**	47.4	11.0
**Age categories (n, %)**		
18–24 y	341	2.3
25–34 y	1488	10.2
35–44 y	3885	26.6
45–54 y	4958	33.9
55–64 y	3161	21.6
65–74 y	712	4.9
75–84 y	77	0.5
85+ y	5	0.0
**Sex (n, %)**		
Male	3872	26.5
Female	10,755	73.5
**Geographic region (n, %)**		
Northeast	2523	17.2
North Central	4071	27.8
South	5139	35.1
West	2811	19.2
Unknown	83	0.6
**Index year (n, %)**		
2007	2440	16.7
2008	3129	21.4
2009	1923	13.1
2010	1729	11.8
2011	1304	8.9
2012	1248	8.5
2013	976	6.7
2014	919	6.3
2015	750	5.1
2016	209	1.4

### Treatment pathways

Among the 14,627 patients with MS who were treated with DMTs, 430 different treatment pathways were noted, indicating that there is a high degree of heterogeneity in treating this disease. Only 38 of these pathways were followed by ≥50 patients and were included in an interactive version of the Sankey diagram (Supplementary material).

The most common first-course treatments (*N* = 14,627) were glatiramer acetate (40.2%), IFN β-1a IM (21.9%), and IFN β-1a SC (14.7%). In contrast, the most common second (*n* = 7510) and third course (*n* = 3882) treatments were glatiramer acetate (32.0 and 32.7%), dimethyl fumarate (15.9 and 17.0%), and IFN β-1a IM (13.8 and 13.3%). Natalizumab, the only infusion DMT, was used by < 2% of patients in any treatment course.

The top 3 pathways were 1 course of glatiramer acetate (19.4%), 1 course of IFN β-1a IM (10.2%), and 1 course of IFN β-1a SC (6.3%). Overall, 30.0% of DMT-treated patients received only glatiramer acetate during the follow-up period. Among these 4384 patients, 64.7% had 1 course of glatiramer acetate, 19.7% had 2 courses, and 15.6% had 3 courses. On average, patients with multiple courses of glatiramer acetate had longer durations of their treatment pathway (3.1, 3.9, and 3.5 years for 1, 2, and 3 courses, respectively) and longer time on drug (3.1, 3.4, and 2.7 years for 1, 2, and 3 courses) compared with those on other DMTs.

Among the 14,627 DMT treated patients, the most common pathways involving treatment switching were from glatiramer acetate to either dimethyl fumarate (*n* = 263 [1.8%]) or fingolimod (*n* = 168 [1.1%]). Compared with patients with a second course of glatiramer acetate, those who switched to an oral DMT (dimethyl fumarate, fingolimod, or teriflunomide) had a longer time on treatment pathway (4.2–4.4 years) and a longer time on drug (3.8–3.9 years). In contrast, patients who switched to a different injectable DMT (IFN β-1a IM or IFN β-1a SC) had a shorter time on treatment pathway (2.8–3.1 years) and a shorter time on drug (2.5–2.9 years) compared with those who switched to a different route of administration.

### Treatment patterns

During the follow-up period of 2–10.5 years, 51.3% of DMT-treated patients with MS had evidence of a second treatment course, and 26.5% had evidence of a third treatment course (Table 2). For all treatment courses and routes of administration, ending a treatment course was most commonly attributed to discontinuation, followed by end of follow-up period and switching. The percentage of patients ending a treatment course due to discontinuation increased with each subsequent treatment course: 50.9% discontinued first course vs 55.6% discontinued second-course vs 57.9% discontinued third course. However, 67.9% of patients who ended their first treatment course and 71.5% who ended their second treatment course due to discontinuation either restarted their prior DMT or switched to a new DMT after a gap in treatment.

**Table 2:** Treatment patterns

	DMT-treated patients		First Treatment Course					
			Oral		Injectable		Infusion	
**Duration of follow-up (mean, SD), mo**	62.3	30.1	51.7	25.1	63.6	30.5	61.5	29.6
**First DMT treatment course**								
**Patients with first DMT course (n, %)**	14,627	100	1583	100	12,796	100	248	100
**Time to first DMT (mean, SD), mo**	5.7	12.7	16.2	22.7	4.1	9.5	18.1	24.4
**Time on first DMT (mean, SD), mo**	28.1	25.9	21.0	17.5	29.0	26.7	27.2	25.4
**First DMT end (n, %)**								
Switch	2450	16.7	140	8.8	2287	17.9	23	9.3
Discontinuation (≥60-day gap)	7447	50.9	741	46.8	6613	51.7	93	37.5
End of follow-up period	4730	32.3	702	44.3	3896	30.4	132	53.2
**Second DMT treatment course**								
**Patients with second DMT (n, %)**	7510	51.3	541	34.2	6898	53.9	71	28.6
**Treatment gap (mean, SD), mo**	5.1	9.3	4.6	6.4	5.2	9.6	5.9	7.7
**Time on second DMT (mean, SD), mo**	15.1	17.4	11.7	11.7	15.3	17.7	13.7	15.3
**Second DMT end (n, %)**								
Switch^a^	899	12.0	42	7.8	845	12.2	12	16.9
Discontinuation (≥60-day gap)^a^	4172	55.6	277	51.2	3856	55.9	39	54.9
End of follow-up period^a^	2439	32.5	222	41.0	2197	31.8	20	28.2
**Third DMT treatment course**								
**Patients with third DMT (n, %)**	3882	26.5	200	12.6	3645	28.5	37	14.9
**Treatment gap (mean, SD), mo**	5.0	7.2	4.7	6.0	5.0	7.3	3.8	5.4
**Time on third DMT (mean, SD), mo**	11.7	13.9	8.8	9.3	11.9	14.1	13.8	15.2
**Third DMT end (n, %)**								
Switch^b^	428	11.0	15	7.5	404	11.1	9	24.3
Discontinuation (≥60-day gap)^b^	2248	57.9	100	50.0	2131	58.5	17	45.9
End of follow-up period^b^	1206	31.1	85	42.5	1110	30.5	11	29.7

^a^Percentage calculated from number of patients with second-course DMT

^b^Percentage calculated from number of patients with third-course DMT

Overall, 51.2% of patients restarted their first treatment course DMT as their second treatment course DMT (Table 3). This included 49.5% of oral DMT users and 51.5% of injectable DMT users but only 28.2% of infusion users. Regardless of the route of administration of their first DMT, patients who switched to a new medication for their second treatment course were most commonly switched to an oral DMT. The mean duration of therapy decreased with each subsequent treatment course (28.1 months for first-line vs 15.1 months for second-line vs 11.7 months for third-line), but the gap between the MS diagnosis and initial treatment or between treatment courses averaged 5–6 months (Table 2).

**Table 3:** Treatment pathways by route of administration

	DMT-treated patients		First Treatment Course					
			Oral		Injectable		Infusion	
**Second DMT treatment course**								
n	7510		541		6898		71	
Route of administration (n, %)								
Oral	2247	29.9	423	78.2	1799	26.1	25	35.2
Injectable	5119	68.2	106	19.6	4993	72.4	20	28.2
Infusion	144	1.9	12	2.2	106	1.5	26	36.6
Restart first DMT (n, %)	3843	51.2	268	49.5	3555	51.5	20	28.2
**Third DMT treatment course**								
n	3882		200		3645		37	
Route of administration (n, %)								
Oral	1234	31.8	149	74.5	1067	29.3	18	48.6
Injectable	2587	66.6	47	23.5	2530	69.4	10	27.0
Infusion	61	1.6	4	2.0	48	1.3	9	24.3
Restart second DMT (n, %)	2325	59.9	115	57.5	2194	60.2	16	43.2

**Fig. 2:**
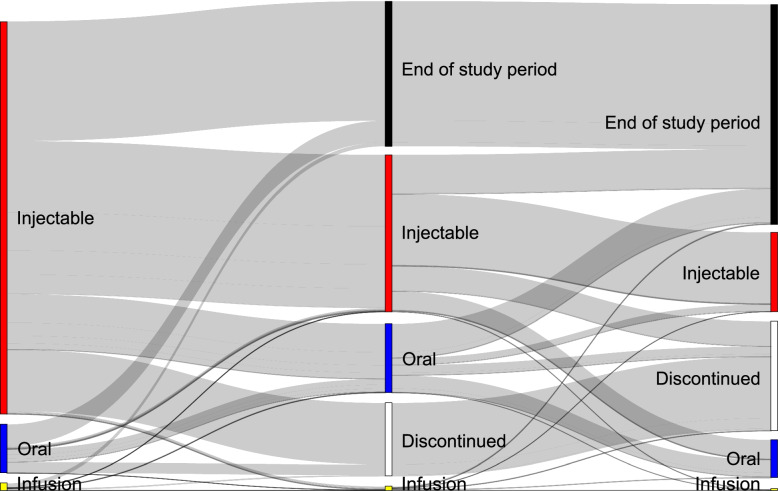
Sankey diagram of treatment pathways by route of administration

During their first treatment course, 87.5% of patients used an injectable DMT, 10.8% used an oral DMT, and 1.7% used an infusion DMT (Table 2). Use of injectable DMTs decreased to 68.2% of patients with the second treatment course and to 66.6% of patients with the third treatment course; whereas use of oral DMTs increased to 29.9% of patients with a second treatment course and 31.8% of patients with the third treatment course (Table 3). Patients who received an injectable DMT as their first treatment course had the highest rates of discontinuation compared with patients who received an oral or infusion DMT as their first treatment course (Table 2). Patients who received an oral DMT as their first treatment course had the lowest rates of switching at the end of each subsequent treatment course. Treatment pathways by use of injectable, oral, and infusion DMT are depicted in Fig. 2 and Supplementary Fig. 1.

## Discussion

In this analysis of treatment pathways, approximately 70% of patients newly diagnosed with MS who initiated treatment with DMTs switched or discontinued from their DMT over a follow-up period of 2–10.5 years. Injectable DMTs in general, and glatiramer acetate in particular, were the most commonly used DMTs over this study period. Oral DMTs were used more commonly as second or third treatment courses, and use of infusion DMTs was uncommon in all treatment courses. The use of oral DMTs in second and third courses was likely influenced by the later approval dates of these medications and by insurance policies that restricted the prescribing options of physicians [12].

Single courses of glatiramer acetate and IFN β-1a were the most common DMT pathways among patients with MS, but more than 400 treatment pathways were observed. Overall, the top 10 treatment pathways were all single courses of the first DMT or repeated courses of the first course DMT following a prolonged interruption. This analysis cannot determine the reason for gaps in therapy but commonly reported reasons include adverse effects, treatment fatigue, perceived lack of efficacy, and practical barriers to compliance such as fear of injections [13, 14]. Changes to insurance plan coverage, such as cost-sharing increases, have also been associated with disruptions in continuous treatment with DMTs [15].

The 2 most common first-course treatments were glatiramer acetate and IFN β-1a IM, and glatiramer acetate and dimethyl fumarate were the most common second- and third-course treatments. In another claims analysis conducted by Kern and Cepeda (2020) in MS patients from 2014 to 2019, glatiramer acetate and dimethyl fumarate were the most common first-line treatments [16]. Our findings that these treatments were most common in later treatment courses are compatible since the current study covers an earlier time period but also overlaps with the study period reported by Kern and Cepeda. Among patients who switched to another DMT, the most common switch was from first course glatiramer acetate to dimethyl fumarate. The same finding was also found in the claims analysis by Kern and Cepeda.

There was some evidence supporting route of administration as a factor influencing treatment patterns. For example, patients who initiated on an oral therapy were less likely to end each treatment course because of switching or discontinuation than patients who initiated on an injectable DMT. Several other studies have examined treatment patterns for oral and injectable DMTs among MS patients in the MarketScan Research databases. One study found that patients initiating on oral DMTs had the highest likelihood of persistence on DMT (66.6%) compared with those initiating on injectable DMTs (58.1%) during a 12-month follow-up period [17]. Another study found that patients who switched to an oral DMT (after initial DMT) had higher persistence rates (60.4%) and also lower odds of having a relapse after switching compared to those who switched to injectable DMTs (46.2% persistence rate), which potentially points to a difference in efficacy among DMTs by route of administration [8]. In our study, when examining the top 38 pathways, patients who switched to an oral DMT after a first treatment course of glatiramer acetate had a longer time on their treatment pathway and a longer time on drug than patients who initiated a second round of glatiramer acetate or who switched to a different injectable DMT. The reasons for this finding are unclear from this analysis as less is known regarding the long-term treatment pathways of newer DMTs (especially the oral forms) due to having less time on the market. A follow-up study comparing treatment patterns by index year may help clarify some of these findings and elucidate potential changes in treatment patterns as newer agents became available for clinical use.

## Limitations

The primary limitation of this study is that not all medications were available at the start of the data collection period, and not all medications were equally available to prescribers because of insurance coverage. In addition, a few medications approved for MS were not included in this analysis owing to their later approval dates or low utilization based on a preliminary analysis of the dataset. As new medications were approved, treatment strategies may have shifted, but those temporal changes would not be visible in the current, aggregated analysis. It is also worth noting that about half of eligible newly diagnosed MS patients did not have any outpatient pharmacy or outpatient medical claims for a DMT during their follow-up period (Supplementary Table 1). It may be the case that some of these patients had less severe disease that did not warrant DMT treatment or that patients may have taken a DMT that was not reported in this study (eg, ocrelizumab, rituximab). This result is comparable to that of another claims analysis that found that 65% of newly diagnosed MS patients were not treated with any DMTs during a minimum 1-year follow-up period [16]. The reasons for non-treatment (as well as reasons for prescribing a type of DMT) are not included in the claims database. Although our study focused on DMT-treated patients and their treatment pathways, the characteristics and management practices of the untreated MS population are worth further exploration.

Other limitations of this study are typical of retrospective administrative claims analyses. Data collected for administrative purposes may not be collected and validated with the same rigor as data collected for other research purposes. Moreover, treatment patterns and pathways were based on filled prescriptions. Patients were assumed to have taken the medications as prescribed; however, this type of analysis cannot confirm whether patients actually took the medications or how much they took. Additionally, reasons for discontinuing or stopping treatment are unknown. Also, the study was limited to only those individuals in the United States with commercial health coverage or private Medicare supplemental coverage. Consequently, the results of this analysis may not be generalizable to patients with MS who have other insurance types, are without health insurance coverage, or are outside the United States. Finally, the current study used a 2-year minimum follow-up period and may have excluded people with less stable health insurance or who died prior to the end of the 2-year minimum follow-up period.

## Conclusions

This study demonstrated a high diversity in treatment patterns among patients newly diagnosed with MS. It was common for patients to have gaps in treatment exceeding 60 days, and the majority of patients restarted their prior DMT after a treatment gap. Thus, treatment cycling occurs frequently among DMT-treated patients with MS and therefore requires more in-depth understanding of current trends among treating physicians.

## Supplementary Information

**Additional file 1.**

## Data Availability

The data that support the findings of this study are available from IBM Watson Health. However, restrictions apply to the availability of these data, which were used under license for this study and are not publicly available.

## Abbreviations

DMT: Disease-modifying therapy
MS: Multiple sclerosis
CNS: Central nervous system
FDA: US Food and Drug Administration
ICD-9-CM and ICD-10-CM: International Classification of Diseases, Ninth and Tenth Revisions, Clinical Modification
IFN: Interferon
PegIFN: Peginterferon
SD: Standard deviation
IM: Intramuscular
SC: Subcutaneous
