# Supplementary material for: Real-world disease-modifying therapy pathways from administrative claims data in patients with multiple sclerosis
Source: BMC Neurol. 2022 Jun 7;22:211. doi: 10.1186/s12883-022-02738-7 (PMC9172015; doi:10.1186/s12883-022-02738-7)
Supplement: Supplementary file 1 — Additional file 1. [file 12883_2022_2738_MOESM1_ESM.docx]

**Supplementary Material**

**Real-World Disease-Modifying Therapy Pathways From Administrative Claims Data in Patients With Multiple Sclerosis**

Robert J. Fox, Rina Mehta, Timothy Pham, Julie Park, Kathleen Wilson, Machaon Bonafede

**Corresponding Author:**
Kathleen Wilson, IBM Watson Health, Cambridge, MA (kawilson@us.ibm.com)

**Supplementary Figure 1.** Sankey diagram of the 38 most commonly observed treatment pathways. Treatment pathways with fewer than 50 patients were excluded from the diagram.

**
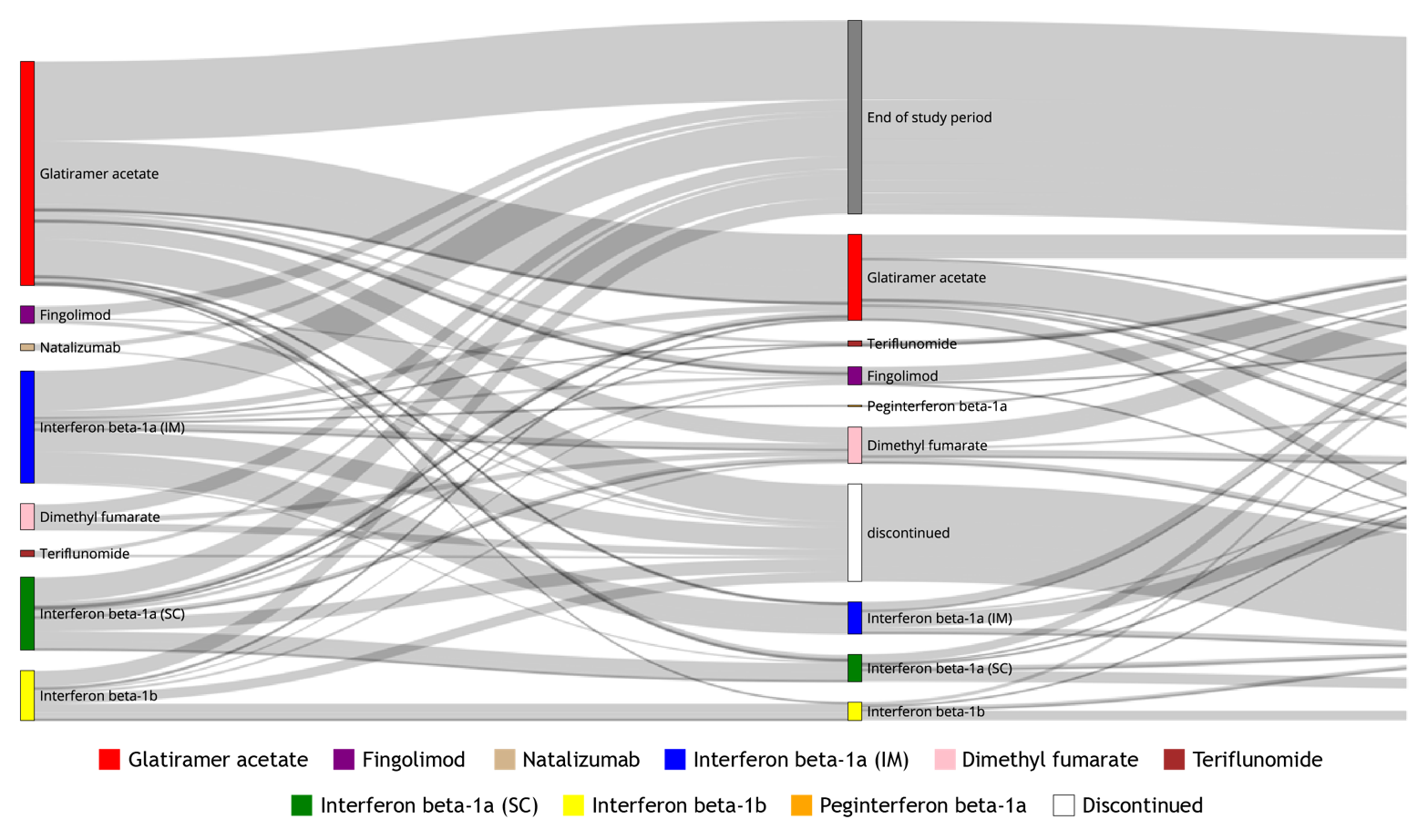
**

**Sankey diagram of multiple sclerosis treatment patterns—variable length post–24 month sample (*double-click icon*):**

**Supplementary Table 1.** Patient characteristics of DMT-untreated patients

|  | **DMT-untreated patients**  **N=15,020** | |
| --- | --- | --- |
| **Age (mean, SD), y** | 53.8 | 14.3 |
| **Age categories (n, %)** |  |  |
| 18–24 y | 370 | 2.5 |
| 25–34 y | 873 | 5.8 |
| 35–44 y | 2684 | 17.9 |
| 45–54 y | 3984 | 26.5 |
| 55–64 y | 3840 | 25.6 |
| 65–74 y | 1981 | 13.2 |
| 75–84 y | 1008 | 6.7 |
| 85+ y | 280 | 1.9 |
| **Sex (n, %)** |  |  |
| Male | 4134 | 27.5 |
| Female | 10,886 | 72.5 |
| **Geographic region (n, %)** |  |  |
| Northeast | 2948 | 19.6 |
| North Central | 4567 | 30.4 |
| South | 4883 | 32.5 |
| West | 2526 | 16.8 |
| Unknown | 96 | 0.6 |
| **Index year (n, %)** |  |  |
| 2007 | 2080 | 13.8 |
| 2008 | 2046 | 13.6 |
| 2009 | 1899 | 12.6 |
| 2010 | 1803 | 12.0 |
| 2011 | 1472 | 9.8 |
| 2012 | 1624 | 10.8 |
| 2013 | 1323 | 8.8 |
| 2014 | 1300 | 8.7 |
| 2015 | 1135 | 7.6 |
| 2016 | 338 | 2.3 |
